# Hypothesis on PTSD Pathophysiology: Role of CRH, Noradrenaline, and Glucocorticoid Receptors in an Amygdala-Centered Closed-Loop System

**DOI:** 10.3390/ijms27146384

**Published:** 2026-07-18

**Authors:** Ilaria Demori, Bruno Burlando

**Affiliations:** Department of Pharmacy, DIFAR, University of Genova, Viale Benedetto XV, 3, 16132 Genova, Italy; bruno.pietro.burlando@unige.it

**Keywords:** sympathetic–adreno–medullary (SAM) system, hypothalamic–pituitary–adrenal (HPA) axis, dynamical systems, central amygdala, basolateral amygdala, locus coeruleus, prefrontal cortex, hippocampus

## Abstract

Post-traumatic stress disorder (PTSD) is a severe condition triggered by traumatic exposure, characterized by symptoms like trauma re-experiencing, avoidance, mood alterations, hypervigilance, and sleep disturbances. While its exact mechanisms remain uncertain, PTSD involves dysregulation across neurobiological systems underlying fear conditioning, threat appraisal, executive control, and stress response. Although research highlights the sympathetic–adreno–medullary (SAM) system and the hypothalamic–pituitary–adrenal (HPA) axis, findings on stress-related mediators remain inconsistent regarding their precise contributions over time. To address this, we propose a hypothetical model viewing PTSD as a multistable system shifting from physiological to pathological steady states. We assume that intense, repeated emotional stress triggers spike activation in the amygdala, driving an amygdala–locus coeruleus loop into a high-activation state via reciprocal excitation, mediated by corticotropin-releasing hormone (CRH) and noradrenaline. This sequentially alters amygdala–hippocampus and prefrontal cortex loops, reinforcing fear expression and impairing extinction. This model is consistent with key features of PTSD, including its higher prevalence among females, increased glucocorticoid receptor sensitivity, the frequently observed hypocortisolism, and the partial efficacy of serotonin and norepinephrine reuptake inhibitor (SNRI) and CRH receptor antagonists. While requiring experimental validation, this framework connects molecular, circuit, and behavioral data to help identify novel interventions for restoring adaptive stress-response dynamics.

## 1. Introduction

Post-traumatic stress disorder (PTSD) is a severe and persistent psychophysiological condition that may develop following exposure to traumatic events. Its core symptomatology includes re-experiencing phenomena (e.g., flashbacks, trauma-related nightmares, and intrusive recollections), cognitive and behavioral avoidance of trauma-associated cues, negative alterations in cognition and mood, and heightened autonomic arousal, manifesting as irritability, sleep disturbances, and hypervigilance. Diagnostic criteria require the persistence of these symptom clusters for at least one month, accompanied by clinically significant distress or functional impairment [1].

Epidemiological data estimate a cross-national lifetime prevalence of PTSD of 3.9% [2]. Women are approximately twice as likely as men to develop PTSD following trauma, even after controlling for differences in trauma exposure and pre-existing psychiatric disorders [3]. These findings suggest that sex-related biological factors may contribute to vulnerability to trauma-related psychopathology.

The pathophysiology of PTSD is complex and involves dysregulation across multiple neurobiological systems, particularly those implicated in fear conditioning, threat appraisal, executive control, contextual processing, and stress response [4]. Upon sensory and emotional processing of real or perceived threats, the amygdala is rapidly activated, triggering the organism’s fear response through downstream engagement of key structures, including the locus coeruleus (LC) and the hypothalamus [1]. In parallel, the prefrontal cortex (PFC) and hippocampus play a critical role in contextual appraisal and top-down modulation of this response [5]. Dysregulation within these interconnected circuits may therefore contribute to the persistence of fear responses and sustained hypervigilance. In line with this framework, functional neuroimaging studies in individuals with PTSD consistently demonstrate reduced activity in the ventromedial PFC (vmPFC), heightened amygdala responsivity [6], and reduced hippocampal volume [7].

PTSD can thus be conceptualized as a maladaptive manifestation of the stress response, in which the tightly interconnected network of amygdala, hippocampus, and PFC fail to realize normal activation and termination of stress-related processes. Given that PTSD arises following exposure to traumatic stressors, considerable attention has been directed toward the two principal effector systems of the stress response: the sympathetic–adreno–medullary (SAM) system and the hypothalamic–pituitary–adrenal (HPA) axis.

Despite extensive investigation, findings regarding stress-related mediators in PTSD remain inconsistent and, at times, paradoxical. This has led to persistent uncertainty regarding the relative contributions of stress hyperactivation, impaired recovery, and their dynamic interaction over time. Accordingly, the role of stress-response dynamics in the pathogenesis of PTSD remains incompletely understood [8].

Following a brief overview of the physiological stress response and current evidence on alterations of the SAM system and HPA axis mediators in PTSD, this paper proposes a revised conceptual framework centered on the dysregulation of the central stress circuitry dynamics. We further argue that a deeper understanding of these dynamic interactions may be essential for the development of novel methodological approaches and the identification of more precise therapeutic targets.

## 2. Physiology of the Stress Response

The stress response is a highly adaptive, dynamic, multi-phased process designed to ensure the survival of the individual in response to environmental challenges. Physical or emotional stressors are processed by a complex circuitry of different, though partially overlapping, brain areas, which detect and appraise the events and trigger a response that integrates body and mind to allow coping strategies [9].

The stereotypical stress response involves the activation of two main effector systems. The SAM system consists of a neural pathway to the adrenal medulla that provides an immediate, hard-wired, fight-or-flight response to a perceived threat. On the other hand, a slower and more sustained response to stress is mediated by the HPA axis, which originates in the brain and elicits an endocrine cascade to the adrenal cortex. Together, these systems form an integrated response: the SAM system provides a fast, transient reaction to immediate challenges, while the HPA axis ensures sustained physiological adjustment to ongoing stress [10].

### 2.1. The SAM System

When a stressor is encountered (e.g., physical danger, emotional shock, or sudden exertion), the amygdala alerts the hypothalamus. In doing so, the basolateral amygdala (BLA), the input region of the amygdala, evaluates sensory information from the cortex and thalamus. Subsequently, the central amygdala (CeA), the output region, becomes activated and sends excitatory projections to the hypothalamic paraventricular nucleus (PVN) and the lateral hypothalamus [11]. In turn, the hypothalamus activates the sympathetic nervous system (SNS) via descending pathways. The hypothalamo–spinal tract involves parvocellular pre-autonomic neurons of the PVN. These neurons send projections, mainly via excitatory glutamatergic drive, to autonomic centers in the intermediolateral cell column (IML) of the thoracic spinal cord, where preganglionic sympathetic neurons are located. The indirect route involves PVN projections to brainstem autonomic nuclei, especially the rostral ventrolateral medulla (RVLM), which then projects down to the same spinal sympathetic neurons [12].

Another pathway through which the CeA activates the SNS involves the pontine LC. The LC receives excitatory, predominantly glutamatergic, inputs from the CeA and is further modulated by amygdala projections releasing corticotropin-releasing hormone (CRH). As the principal brain source of noradrenaline (norepinephrine), the LC projects widely to cortical and subcortical regions, as well as to the spinal cord, including the IML. At this level, noradrenergic inputs modulate the excitability of sympathetic preganglionic neurons, facilitating the effects of descending glutamatergic drive from the PVN and RVLM [13]. In addition, the PFC exerts top-down regulatory control over LC activity through a combination of direct glutamatergic projections and indirect pathways involving inhibitory interneurons and brainstem nuclei [14]. Dysfunction of this PFC-mediated control may contribute to sustained LC activation, sympathetic hyperactivity, and impaired emotional regulation.

Descending pathways from the PVN and RVLM ultimately lead to the activation of the adrenal medulla, a modified nerve ganglion, inducing the release into the bloodstream of catecholamines, namely adrenaline (epinephrine), accounting for roughly 80% of the total, and noradrenaline, about 20% [15]. Once released, these hormones bind to adrenergic receptors throughout the body, causing, among others, increased heart rate and force of contraction, redirection of blood flow from the skin and digestive tract to skeletal muscles and the brain, dilation of the airways to maximize oxygen intake, mobilization of glycogen into glucose in the liver to provide immediate fuel, and dilation of the pupils (mydriasis) increasing light entry and retinal illumination [15].

The rapid fight-or-flight activation is quickly counteracted by the rest-and-digest response of the parasympathetic nervous system once the threat has passed. Adrenaline and noradrenaline are eliminated through reuptake or rapid degradation by enzymes such as catechol-O-methyltransferase (COMT) and monoamine oxidase (MAO), allowing the body to return to homeostasis [16].

A regulatory role of these responses is also played by the mPFC via projections to brainstem pre-autonomic nuclei. In general, the prelimbic (PL) mPFC inhibits the SNS, whereas the infralimbic (IL) mPFC induces its activation in response to acute stress [17].

### 2.2. The HPA Axis

The functioning of the HPA axis is conventionally assumed to begin with specific neurons located in the PVN, which release CRH into the median eminence and then, through the portal circulation, to the anterior pituitary. There, CRH stimulates corticotroph cells to release adrenocorticotropic hormone (ACTH), which travels via the systemic circulation to the adrenal glands, where it stimulates the synthesis and secretion of corticosteroids (mainly cortisol in humans). These, in turn, regulate their own production by exerting negative feedback on both the hypothalamus and the pituitary, inhibiting CRH neurons and corticotroph cells [18].

Like many endocrine activities, the HPA axis exhibits a rhythmic pattern that is independent of stress responses and consists of ultradian (about one pulse per hour) and circadian components [19]. Circadian and ultradian components of HPA activity are mediated by neuroendocrine mechanisms acting on the PVN. The circadian rhythm peaks in the morning with the cortisol awakening response (CAR), declines throughout the day, and bottoms at about midnight. The fluctuation is primarily regulated by the suprachiasmatic nucleus (SCN), the principal site of the biological clock driven by the light–dark cycle [20]. The ultradian rhythm, which overlaps with the circadian one, has been explained as the result of a delay in the negative feedback of the HPA axis [21], consistent with the behavior of delayed negative-loop dynamical systems with cyclic attractors. However, in the PVN of mice, a CRH-neuron pacemaker has also been identified that can be activated by a glutamatergic pathway originating from the mPFC, thereby promoting social avoidance, threat memory, and an imbalance between fear acquisition and fear extinction [22]. More generally, the mPFC does not project directly to the PVN but instead sends projections to adjacent regions containing GABAergic neurons that exert regulatory control over PVN activity [23]. Consistent with this organization, a dopamine-dependent prefrontal cortex–peri-PVN–PVN circuit has been shown to suppress hypothalamic CRH neuron activity and prevent the emergence of anxiety-like behaviors [24].

Cortisol exerts its pleiotropic effects upon binding to high-affinity mineralcorticoid receptors (MRs) and low-affinity glucocorticoid (GC) receptors (GRs). The latter are widely expressed throughout peripheral tissues and in the CNS, where they play a dominant role in mediating the negative feedback control of the HPA axis. Because of their relatively low affinity, these receptors become engaged only when circulating GC levels rise substantially, such as during the circadian peak or under stress conditions. In addition to being expressed in pituitary corticotrophs, GRs exhibit widespread distribution throughout the hypothalamus, amygdala, PFC, and hippocampus, the latter being considered a key brain region to exert downregulation of cortisol production [25]. Activation of GRs triggers long-term genomic changes within target tissues [26], leading to pleiotropic effects, including regulation of energy metabolism (hyperglycemic and catabolic effects), enhanced cardiovascular function, regulation of cognition and mood, and immunomodulation [27].

In cases of acute stress, the SNS and the HPA axis are immediately activated, leading to increases in adrenaline, noradrenaline, and cortisol characterized by different time constants. Noradrenaline is released immediately via two pathways, from sympathetic nerve endings onto organs, and from the adrenal medulla (about 20% of gland secretion) into the blood. Neurotransmitter spikes occur within milliseconds at the nerve endings, while measurable plasma concentrations rise within 1 to 2 s, reaching peak levels within 30 to 60 s [28]. Adrenaline is released mainly by the adrenal medulla (about 80% of gland secretion), and its plasma concentrations rise noticeably within 1 to 2 s of the stressor, reaching maximum peak levels within about 45 to 90 s [29]. Cortisol levels peak within 30 min after the onset of a stressor and return to baseline levels 60–90 min after the stressor ends [30].

Conversely, chronic stress, characterized by prolonged activation of the HPA axis over days or months, leads to an impairment of the HPA rhythm and feedback control, resulting in conditions of constant hypercortisolism or even hypocortisolism [31]. Hypercortisolism has been associated with several diseases that are triggered or worsened by chronic stress, including obesity, dysmetabolism, depression and even cancer. However, several data support the hypothesis that in some instances, an initial stress-induced rise in cortisol levels is followed by a reduction, and hypocortisolism has been associated with conditions such as chronic fatigue, rheumatoid arthritis, and PTSD [32].

## 3. Dysregulation of the Stress Response in PTSD

### 3.1. The SAM System

In PTSD, catecholamines are frequently reported to be altered, with several studies indicating increased plasma levels [33]. This pattern is thought to stem from persistent activation of the SNS, even in the absence of a threat [34]. While the SAM system is generally adaptive in the context of acute stress, chronic stress exposure may lead to sustained activation and dysregulation. Prolonged activation has been associated with a range of stress-related psychiatric disorders, including anxiety, depression, and PTSD, and the role of catecholamines and their adrenoceptors in these conditions has been extensively investigated [35]. A meta-analysis comparing catecholamine plasma and/or urinary levels in PTSD patients and controls showed no significant differences in dopamine concentrations, and higher, though not statistically significant, levels of adrenaline, whereas noradrenaline levels were significantly elevated [36]. These findings suggest that noradrenaline emerges as the most consistently altered catecholamine in PTSD.

This observation is consistent with the well-established interplay between the amygdala and the LC, the main source of central noradrenergic projections. Experimental studies in mice have demonstrated that LC noradrenergic fibers stimulate neuronal activity in the BLA via β-adrenergic receptors, thereby enhancing LC-induced anxiety behavior [37] and impairing extinction learning [38,39]. Reciprocally, the amygdala activates LC neurons during a stress response, supporting the existence of a positive feedback loop between these two regions. Such a loop may contribute to the emergence of a self-sustained circuit of heightened arousal and impaired fear regulation, independent of external threats. This could explain why noradrenaline is consistently high in PTSD patients compared to other catecholamines, suggesting potential therapeutic applications [40].

Experimental models also support the putative role of noradrenaline in sustaining amygdala hyperactivity. Mice exposed to repeated predator exposure have developed CRH-induced overexpression of α1-adrenergic receptors in excitatory neurons of the BLA, enhancing sensitivity to noradrenergic inputs and amplifying startle responses reminiscent of PTSD [41]. In auditory fear-conditioned rats, β-adrenergic stimulation in the amygdala enhances reconsolidation and persistence of fear memories [42].

The PFC is another target for LC-derived noradrenaline. Activation of postsynaptic α1-adrenergic receptors has been associated with stress-induced impairment of prefrontal cortical function, promoting heightened arousal and reduced cognitive flexibility. In contrast, under optimal arousal conditions, moderate levels of noradrenaline engage α2-adrenergic receptors, located both presynaptically in the LC and postsynaptically in the PFC. These receptors mediate inhibitory feedback and support executive control processes, including working memory and attentional regulation [43].

In synthesis, PTSD is associated with enhanced SNS reactivity and hyperarousal, rather than consistent tonic elevation of SAM axis activity. While noradrenaline levels are often increased, adrenaline levels show no reliable baseline elevation, and the most robust finding is exaggerated sympathetic responses to stress-related or conditioned stimuli [36,44]. Moreover, in PTSD, the combined activation of α1 and β receptors, together with insufficient α2-mediated feedback, may promote both heightened arousal and the strengthening of maladaptive fear memories. These findings have suggested a central role for noradrenaline in the pathogenesis of PTSD [45].

### 3.2. The HPA Axis

Noradrenergic dysregulation in PTSD is closely intertwined with alterations of the HPA axis, the other major effector system of the stress response. Despite considerable heterogeneity in the literature, alterations of HPA axis function have frequently been reported in PTSD. Meta-analyses indicate that cortisol findings vary according to biological matrix, time of sampling, trauma type, and psychiatric comorbidities, although many studies report reduced basal cortisol levels in saliva, urine, or serum of PTSD patients [46,47,48,49]. This condition is characterized by apparently paradoxical features: although elevated CRH levels have been reported in both the cerebrospinal fluid and plasma of individuals with PTSD [50,51], a blunted ACTH response and a reduction in cortisol production have been documented following the dexamethasone suppression test in PTSD compared to controls [33].

This situation is ascribed to an enhanced sensitivity of glucocorticoid-mediated negative feedback [52,53,54]. Consistently, higher GR expression has been found in PTSD patients [55,56]. The GR normally forms a heterocomplex with the chaperone FK506-binding protein 5 (FKBP5), which regulates ligand binding and translocation of the GR to the nucleus for genomic activity, such that lower FKBP5 activity corresponds to higher GR sensitivity. Several single-nucleotide polymorphisms in GR- and FKBP5-related genes have been associated with PTSD risk [57,58]. The *NR3C1* GR gene, exon 1F (GR-1F), has been found to display lower promoter methylation in peripheral blood mononuclear cells of PTSD patients, along with higher GR expression [59]. Downregulation of FKBP5 has been observed in trauma survivors [60], while a positive correlation between *FKBP5* gene methylation and PTSD symptoms has also been reported [61]. *FKBP5* genetic variants, combined with childhood abuse, appear to increase the risk of PTSD development in adulthood [62]. Together, these findings suggest that enhanced GR sensitivity may represent a more robust biological feature of PTSD vulnerability than absolute circulating cortisol levels.

An unresolved issue in the endocrine pattern of PTSD is the presence of high cerebrospinal and plasma CRH levels, which are not correlated with the normal-to-low ACTH and the low cortisol levels [50].

This apparently paradoxical pattern could be explained by the divergence between the responses of the CeA and the PVN to GCs during acute stress versus the pathological condition of PTSD. Rodent models demonstrate that GCs increase CRH expression in the CeA while reducing it in the PVN. [63]. Studies in mice have shown that elevated CRH release from the CeA increases anxiety-like behavior [64], a mechanism supported by evidence that GR deletion in the CeA suppresses conditioned fear [65]. Complementarily, fear induction directly elicits CeA activation and subsequent CRH production. [66]. Conversely, PVN CRH and the downstream HPA axis are suppressed in PTSD due to the opposing, negative feedback response of PVN GRs to GCs [67]. Extrapolating these data to humans suggests that the high central CRH levels observed in PTSD patients may stem from persistent CeA hyperactivity, whereas the systemic HPA axis would be downregulated by heightened negative feedback driven by excessive GR sensitivity.

Based on the above data, low cortisol would not be a risk factor for PTSD but rather a consequence. The innate tendency to develop the disease would reside in the presence of specific variants of the *FKBP5* and *NR3C1* genes, possibly combined with early traumatic events in childhood. Moreover, although PTSD patients frequently exhibit low cortisol levels, they are more responsive to cortisol [68], which could stimulate higher CRH production in the amygdala, possibly due to increased GR activity. Such a positive feedback loop in the amygdala would therefore contribute to preventing fear extinction and to sustaining pathological fear and anxiety states.

## 4. Molecular Signaling and Pharmacological Implications

Antidepressants, anticonvulsants, antipsychotics, benzodiazepines, and opioids currently represent the pharmacological classes most frequently prescribed to patients with PTSD, often in the context of multiple off-label treatment strategies that remain insufficiently investigated [69,70]. Although these agents may alleviate specific symptom domains associated with PTSD, none has demonstrated consistent efficacy in achieving full remission of the disorder. In 2017, a Letter to the Editor published in Biological Psychiatry emphasized the urgent need for substantial advances in the psychopharmacological treatment of PTSD [69]. This persistent therapeutic gap is likely attributable, at least in part, to the incomplete understanding of PTSD pathophysiology, which continues to hinder the identification and development of novel therapeutic targets.

### 4.1. Serotonergic Signaling and Pharmacotherapy

Serotonin (5-HT) is primarily produced by the forebrain raphe nuclei. From here, serotonergic projections project widely throughout the brain, reaching the amygdala, hippocampus, and PFC, thereby playing a fundamental role in stress responses, emotional regulation, threat appraisal, anxiety, extinction learning, and resilience to adversity [71]. Conversely, during stressful conditions, the expression of 5-HT2A receptors, mediating excitatory actions, increases, and is thought to promote neural plasticity for threat memory and avoidance. However, in traumatic stressful situations, the action of 5-HT can become excessive, being correlated to strong and persistent associations between environmental cues and stress, thus overpowering signals of safety and eventually contributing to the symptoms of PTSD [72].

The role of 5-HT in PTSD is best conceptualized as predominantly modulatory, contributing to the regulation of stress-related neural circuitry rather than acting as the primary driver of the disorder. The serotonergic and noradrenergic systems are highly interconnected and exert reciprocal modulatory influences through extensive anatomical and functional interactions. Serotonergic neurons originating in the dorsal and median raphe nuclei receive noradrenergic inputs from the LC, while serotonergic projections, in turn, regulate the activity of noradrenergic neurons through multiple serotonin receptor subtypes [73]. This bidirectional interplay contributes to the regulation of arousal, stress responsiveness, emotional processing, and cognitive function.

In PTSD, hyperactivity of the LC–noradrenergic system interacts with altered serotonergic neurotransmission, resulting in excessive noradrenergic signaling that enhances vigilance, facilitates the consolidation of traumatic memories, and promotes hyperarousal, whereas serotonergic dysfunction is associated with impaired fear extinction, emotional dysregulation, and increased anxiety [74]. The dynamic interaction between these systems likely contributes to several core PTSD symptoms and may partly explain the therapeutic efficacy of pharmacological treatments targeting serotonergic and/or noradrenergic neurotransmission.

Selective serotonin reuptake inhibitors (SSRIs) and serotonin and norepinephrine reuptake inhibitors (SNRIs), as well as the 5-HT2A agonist 3,4-methylenedioxymethamphetamine (MDMA), are pivotal therapeutic tools in PTSD treatment [75]. However, although effective in reducing symptom severity, clinical tests have shown that they are not able to achieve PTSD remission [76]. It has been proposed that these drugs exert their therapeutic effects by acting on reciprocal projections established by the amygdala with the LC, PFC, and hippocampus. These mechanisms would include different 5-HT receptors, including 5-HT1A, 5-HT2A, and 5-HT2C, enhancing the learning of safety signals and inducing desensitization by prolonged activation, potentially alleviating maladaptive changes linked to fear learning. MDMA may also engage serotonergic and hormonal systems to induce a controlled stress-like state that enables patients to reprocess traumatic memories and engage more effectively in therapy [72]. In summary, although serotonergic signaling can modulate fear expression in a receptor- and circuit-specific manner, PTSD is not characterized by a simple increase in 5-HT levels. Rather, SSRIs, SNRIs, and MDMA exert therapeutic effects through long-term adaptations in serotonergic systems, recovering prefrontal control over limbic circuits and facilitating fear extinction [77].

### 4.2. Adrenergic Signaling and Pharmacotherapy

The α1-adrenergic receptor antagonist prazosin is frequently administered as an adjunctive treatment to SSRI/SNRI therapy because of its efficacy in reducing sleep disturbances and trauma-related nightmares in patients with PTSD. The underlying mechanisms are thought to involve modulation of rapid eye movement (REM) versus non-REM sleep architecture, as well as attenuation of CRH activity [78].

Similarly, the non-selective β-adrenergic receptor antagonist propranolol has been shown to disrupt memory reconsolidation processes within the amygdala in animal models, a mechanism potentially relevant to the retrieval and emotional salience of traumatic memories in PTSD [79]. In clinical settings, propranolol has demonstrated modest efficacy in reducing hyperarousal symptoms and has also been investigated as a strategy for secondary prevention following trauma exposure [80].

At the presynaptic level, central α2-adrenergic receptors function as inhibitory autoreceptors regulating noradrenergic tone through negative feedback mechanisms. Pharmacological activation of these receptors by clonidine and guanfacine has been associated with improvements in hyperarousal, hypervigilance, sleep disturbances, exaggerated startle responses, and trauma-related nightmares in patients with PTSD [81].

### 4.3. HPA Axis–Targeted Pharmacotherapy

In an effort to emulate the modulatory effects of cortisol on excessive trauma-related memory retrieval and reconsolidation processes, hydrocortisone has been investigated as a potential therapeutic option for PTSD, with evidence suggesting more favorable outcomes in preventive or early post-trauma interventions compared with chronic administration [82].

GR modulation via selective antagonism, such as with mifepristone, has been shown to increase circulating cortisol and ACTH levels; however, clinical studies have not demonstrated consistent therapeutic benefits in PTSD [70]. Conversely, pharmacological attenuation of CRH signaling through CRH receptor 1 (CRHR1) antagonists appears more promising. In this context, the CRHR1 antagonist GSK561679 has been reported to reduce PTSD-related anxiety responses and fear-potentiated startle in experimental paradigms [83]. Emerging evidence also suggests the potential relevance of CRHR2 signaling. CRHR2-expressing neuronal populations in the bed nucleus of the stria terminalis (BNST) have been implicated in susceptibility to PTSD-like behavior [84]. Intranasal administration of the CRHR2-specific agonist urocortin 3 has been shown to ameliorate anxiety-like behavior in a PTSD animal model [85].

In addition, neuroactive steroids have received increasing attention as potential therapeutic agents for PTSD. Research has documented reduced levels of the GABAergic allopregnanolone and its stereoisomer pregnanolone in the cerebrospinal fluid of both women and men with PTSD [86,87]. Low neurosteroid levels in PTSD are attributable to impaired biosynthesis, resulting from reduced expression or enzymatic activity of 3α-hydroxysteroid dehydrogenase (in women) or 5α-reductase (in men). These deficits are associated with increased risk for PTSD, more severe symptomatology, greater clinical chronicity, and reduced responsiveness to specific treatments [88]. Converging preclinical and clinical evidence indicates that allopregnanolone and related compounds may restore GABAergic inhibitory tone, modulate CRH signaling, and normalize stress-induced alterations in neural excitability [88,89,90].

Evidence has also underscored the predictive relevance of epigenetic modifications, particularly DNA methylation patterns in genes such as *NR3C1* and *FKBP5*, which may act as predisposing factors for PTSD development [91]. These findings emphasize the necessity of incorporating epigenetic variability into models of treatment response and therapeutic stratification in PTSD [70].

## 5. A Hypothetical Model of PTSD Pathogenesis

The pathogenesis of PTSD is still incompletely understood, and only hypothetical models can currently be proposed due to the limited understanding of its underlying mechanism. However, available evidence indicates that such a mechanism must involve, at some level, the modulation of stress response processing.

**Figure 1:**
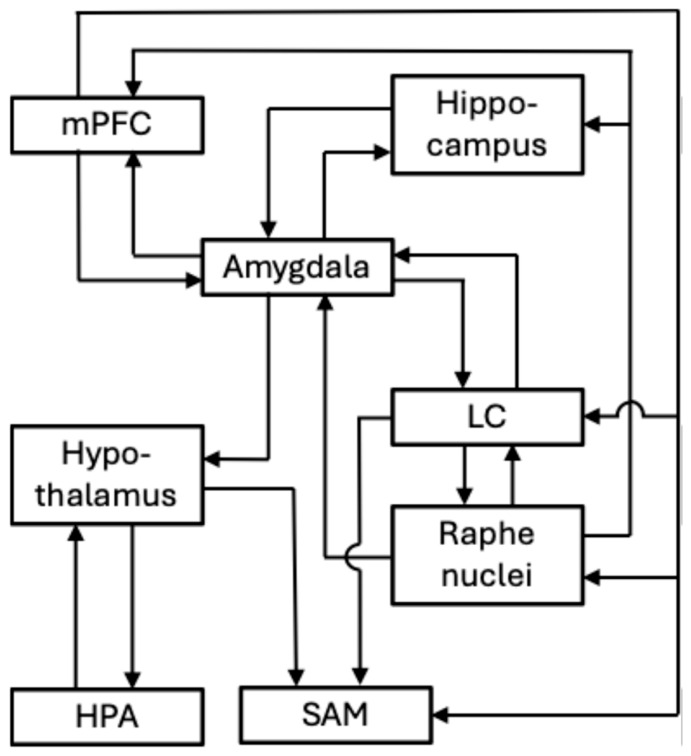
General representation of the interactions among the main neural and endocrine systems involved in the stress response processing. HPA, hypothalamic–pituitary–adrenal axis; LC, locus coeruleus; mPFC, medial prefrontal cortex; SAM, sympathetic–adreno–medullary system. Lines with arrowheads indicate the direction of pathways, regardless of their excitatory or inhibitory nature.

In the preceding overview, we have considered the principal neurobiological systems implicated in stress processing that have been investigated regarding PTSD pathophysiology and are now summarized in Figure 1.

Although these findings highlight the remarkable complexity of PTSD neurobiology, they do not readily identify the minimal mechanism responsible for the emergence and persistence of the disorder. Therefore, rather than attempting to reproduce the entire stress-response network, our hypothesis introduces an alternative rationale, outlined below.

### 5.1. Theoretical Background for Model Development

We propose a possible mechanism building on a set of our previous modeling of physiological and pathogenic processes (see, for instance, [92,93]). The primary objective is to develop a model that, without claiming to fully reconstruct biological reality, provides a useful interpretative framework for understanding and potentially intervening in the disease. The general idea is to describe living organisms as dynamical systems characterized by regular attractors, which account for the high predictability of physiological processes. Within this framework, phenotypic or functional changes, such as those occurring in pathological states, can be interpreted as transitions between distinct equilibrium states generated by multistable systems.

Different studies have described psychiatric disorders by using dynamical systems modeling [94]. Specifically, this approach has also been used for a data correlation analysis between PTSD, depression symptoms, and suicidal ideation, concerning military personnel with a PTSD diagnosis [95]. However, we assume that diseases are dynamical systems dominated by multistable-attractor landscapes. Therefore, loop-arranged interaction networks should be the best models to analyze their behavior and explore management strategies [96]. Based on these assumptions, we hypothesize that PTSD pathogenesis arises from an interconnected network of positive feedback loops, a configuration typically associated with multistable dynamics. Such a system must be grounded in brain regions consistently implicated in the onset and progression of the disorder.

### 5.2. PTSD Model Formulation

According to the literature, the most consistently reported biological hallmarks of PTSD include elevated CRH and noradrenaline levels, particularly in cerebrospinal fluid and plasma, together with increased GR activity. Other mediators associated with PTSD, namely adrenaline, dopamine, 5-HT, ACTH, and cortisol, show greater variability and lower consistency across studies. As previously discussed, elevated CRH and noradrenaline levels can be associated with the activity of the CeA [64] and the LC [36], respectively. Experimental evidence suggests that these two brain regions are connected through reciprocal excitatory interactions [40], which can therefore be conceptualized as a positive loop.

Moreover, available data, mainly acquired in rodent models, show other main loops established between the BLA and the ventral hippocampus (VH) and the PL and IL areas of the vmPFC. Evidence indicates that the BLA establishes reciprocal-inhibition loops with the VH and IL, whereas a reciprocal-excitation loop is established with the PL [97,98,99]. All these loops can be classified as positive, allowing for bistable behavior. However, in the reciprocal-inhibition loops, the bistable behavior consists of alternating high and low activities of the mutually interacting agents, whereas in the reciprocal-excitation loop, bistability consists of either common activation or depression.

The importance of these loops is highlighted by the well-assessed role of the system comprising the PFC, the amygdala, and the hippocampus in modulating the formation, retrieval, and update of fear and extinction memories [99]. In addition, a reciprocal inhibition model has been proposed based on the assumption of alternating dominance between the amygdala and the vmPFC. This model predicts that when the amygdala dominates, patients display an attentional bias toward threat and manifest re-experiencing symptoms [97].

**Figure 2:**
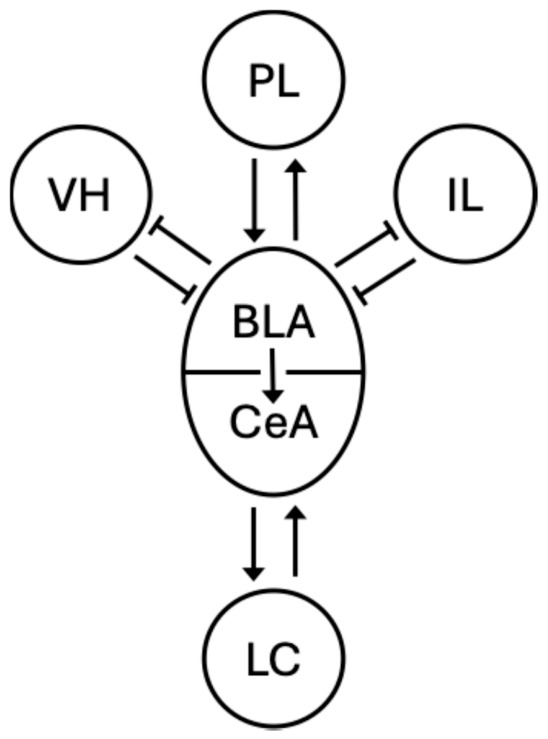
Schematic representation of the proposed pathogenic model of PTSD based on an interconnected loop system (see text for functioning details). BLA = basolateral amygdala; CeA = central amygdala; LC = locus coeruleus; PL = prelimbic cortex; IL = infralimbic cortex; VH = ventral hippocampus. Lines with arrowheads = activations; lines with hammer-endings = inhibitions.

Synthesizing these notions, we hypothesize that under conditions of intense and repeated emotional stress, spike activation of the BLA and CeA drives the amygdala–LC loop toward a high-excitatory equilibrium point. Such a transition would result in a cascade effect on other loop circuits involved in fear expression and extinction. Hence, following a traumatic stress experience, given the upward pull of the amygdala–LC system, the BLA/PL loop could transition to high activity, thereby strengthening amygdala activation. Conversely, the other circuits would shift to high-BLA/low-IL and high-BLA/low-VH stationary states, weakening the control exerted by the IL and VH on amygdala activation, and consequently potently hindering fear extinction (Figure 2).

As stated above, our PTSD model assigns a pivotal role to the amygdala–LC loop through CRH and noradrenaline secretion. Under this view, the wide variable and even counterintuitive patterns observed in the secretion of adrenaline, dopamine, 5-HT, ACTH, and cortisol can be explained by their positions in the cascade of events triggered by the core mechanism. This perspective aligns with our general idea that diseases consist of primary, specific, and predictable mechanisms, which provide the basis for classifying pathological conditions, followed by a panel of cascade events. These subsequent events account for the sometimes highly complex and varied symptomatology observed across patient populations.

Considering the top palliative effects of SSRIs on PTSD symptoms, a further relevant consideration is that the proposed amygdala–LC feedback loop may provide a mechanistic link between the core circuit dynamics and the serotonergic modulation of stress-related behavior. CRH has been shown to influence the activity of serotonergic neurons in the dorsal raphe nucleus, with effects that depend on receptor subtype signaling and stress intensity [100,101]. Consequently, serotonergic dysregulation can be interpreted as a downstream consequence of sustained activation of the core stress circuitry, further supporting the view that serotonergic pharmacological interventions primarily act by reshaping the stability of the pathological attractor state rather than by directly suppressing its core generating mechanism. This interpretation is also consistent with the well-known delayed therapeutic effects of SSRIs and SNRIs, which are thought to reflect progressive neuroplastic adaptations and facilitation of fear extinction rather than acute modulation of synaptic serotonin levels [102].

## 6. Strengths and Limitations of the Model

The main strength of the proposed model lies in its dynamical formulation of PTSD pathogenesis, whereby pathological states emerge as attractors generated by interconnected positive feedback loops involving key stress-related brain regions, with CRH and noradrenaline playing central roles. This representation provides a unified mechanistic interpretation of heterogeneous neurobiological findings and links molecular-level dysregulation to large-scale circuit dynamics. Within the present framework, variability in cortisol measures is not unexpected, since circulating cortisol represents a downstream endocrine readout of the pathological network state rather than the primary determinant of that state.

Furthermore, the model is consistent with known vulnerability factors for PTSD, particularly increased GR sensitivity, which may arise from genetic polymorphisms or early-life trauma and has been associated with the hypocortisolemic profile observed in many patients [33]. Enhanced GR activity could strengthen the proposed pathogenic mechanisms through a possible inversion of cortisol feedback signaling. Specifically, it has been suggested that cortisol feedback may shift from the canonical HPA axis negative feedback control toward a positive feedback loop on the CRH-producing neurons within the CeA [67]. Hence, such an inversion would further sustain the pathogenic transition in the amygdala–LC circuit.

An important feature of the proposed model is its ability to accommodate well-established sex differences in stress-related neurocircuitry and PTSD vulnerability. Converging evidence suggests that females’ increased susceptibility to PTSD is supported by sex-dependent regulation of both CRH signaling and LC physiology. At the receptor level, female rodents show enhanced CRHR1 signaling in both amygdala and LC neurons, increased CRH sensitivity, and reduced stress-induced CRHR1 internalization compared with males [103,104]. At the circuit level, LC shows age- and sex-dependent changes in the interaction with corticosterone following acute stress [105]. Moreover, female rodents exhibit higher intrinsic LC excitability under CRH stimulation [103], resulting in a more sustained noradrenergic response relative to males.

Within the framework of the present hypothesis, these findings do not imply a different circuit architecture, but rather sex-dependent modulation of its dynamical parameters at multiple nodes of the loop. Specifically, enhanced CRH/CRHR1 sensitivity together with increased LC excitability are predicted to synergistically increase the effective gain of the amygdala–LC positive feedback loop in females. This higher gain would favor the emergence and stabilization of a pathological attractor state characterized by persistent amygdala hyperreactivity and impaired extinction, thereby providing a mechanistic account of sex differences in PTSD risk and persistence.

In addition, the partial efficacy of existing pharmacological treatments may reflect their ability to modulate some components of the model, particularly noradrenergic signaling and CRH-mediated pathways. In this regard, emerging CRHR1 antagonists may act by attenuating one of the core positive feedback drivers of the proposed system, thereby contributing to a partial restoration of adaptive stress-response dynamics. Similarly, the effects of GABAergic neurosteroids may be conceptualized as indirectly reducing the gain of the proposed amygdala–LC positive feedback loop. In this regard, neurosteroid concentrations exhibit physiological fluctuations in females across the menstrual cycle and major reproductive life stages, including pregnancy, the postpartum period, and menopause [106]. Consistent with the female prevalence of PTSD and the well-known interplay between the HPA and HPG axes [107], such fluctuations may transiently modulate inhibitory control over amygdala-driven stress circuits, thereby influencing the effective gain of the amygdala–LC positive feedback loop.

However, the proposed hypothesis has not yet received direct experimental validation and therefore remains primarily conceptual. Its validity rests on consistency with known PTSD-associated alterations, including elevated CRH and noradrenaline levels, altered cortisol dynamics, and increased GR activity, as well as its ability to integrate these findings into a coherent systems-level framework. A major limitation concerns the biological instantiation of model variables and parameters, which cannot be derived from any current theory and must therefore be inferred from empirical and often indirect evidence. Moreover, most circuit-level assumptions are based on preclinical studies, limiting direct translational certainty. Although widely used, preclinical models do not fully recapitulate the complexity of psychiatric disorders such as PTSD. Notably, sex differences are often underrepresented, despite the higher risk of PTSD observed in women. Much of the preclinical evidence supporting amygdala–LC circuitry, CRH signaling, and fear conditioning mechanisms has been derived from studies conducted in male rodents [108]. This represents a significant limitation of the current knowledge base and constrains the direct generalizability of circuit-level findings across sexes. In accordance with current recommendations on Sex as a Biological Variable (SABV, NOT-OD-15-102, https://orwh.od.nih.gov/sex-as-biological-variable (accessed on 25 June 2026)), the present hypothesis interprets these male-derived circuit mechanisms as a framework upon which sex-dependent modulatory processes are superimposed.

Overall, both the strengths and limitations of the present framework reflect the current state of theoretical and experimental knowledge in this field. Hence, the explanatory and predictive value of the model remains contingent on future experimental validation.

## 7. Future Perspective

Future work should focus on translating the proposed framework into a quantitative dynamical model incorporating presumed key PTSD-related mediators, including CRH, noradrenaline, cortisol, and GR signaling. This would allow formal analysis of stress-related network dynamics using systems of differential equations.

This approach allows for the exploration of the system’s behavior in phase space where, for fixed parameter values, the variation in variables over time traces trajectories driven by vector fields. In multistable systems, these fields point toward the centers of different basins of attraction where equilibrium points (steady states) reside. Concurrently, subjecting the model to bifurcation analysis highlights those parameters that, by changing in value, reshape the landscape of basins of attraction in the phase space. This can shift the system, for example, from a monostable to a bistable regime, opening the way to changes in the functional state, such as the pathogenic process.

In this context, the mathematical tools are well-established within the fields of Systems and Control Theory. Conversely, the mapping of mathematical model terms to biological counterparts cannot be deduced from any current theory. Hence, the definition of a suitable model, i.e., the proper assignment of variables and parameters to different biological entities, relies on the intuition of specialists with biomedical expertise and must be validated by the model’s ability to identify effective interventions for treating the disease.

In our research, this phase of analysis will be developed in a forthcoming study, where we will investigate the dynamics of the proposed PTSD pathogenesis model to identify potential critical targets for novel therapeutic strategies. In this way, we aim to establish a theoretical foundation for gaining control over the “engine” of the disorder and identifying effective treatments capable of shifting a patient toward a less severe or completely healthy state.

## 8. Conclusions

PTSD can be conceptualized as a disorder of stress-system dysregulation in which persistent pathological states emerge from maladaptive interactions within interconnected neural and endocrine networks. In this work, we propose a mechanistic framework in which key stress-related brain regions and mediators, including CRH, noradrenaline, and GR signaling, contribute to the formation of positive feedback loops capable of generating multistable dynamics and stable pathological attractor states.

Within this perspective, symptoms of PTSD may be interpreted as the behavioral and physiological expression of a system trapped in a dysregulated equilibrium, maintained by reinforcing circuit and neuroendocrine interactions. This framework provides a unifying systems-level interpretation of heterogeneous findings and suggests that disease persistence may arise from state stability rather than solely from ongoing external stress exposure.

Although still hypothetical and requiring experimental validation, this model offers a formal basis for linking molecular, circuit, and behavioral levels of analysis and may help identify novel intervention points aimed at restoring adaptive stress-response dynamics.

## Data Availability

The original contributions presented in this study are included in the article. Further inquiries can be directed to the corresponding author.

## Abbreviations

The following abbreviations are used in this manuscript:

ACTH	adrenocorticotropic hormone
BLA	basolateral amygdala
BNST	bed nucleus of the stria terminalis
CAR	cortisol awakening response
CeA	central amygdala
COMT	catechol-O-methyltransferase
CRH	corticotropin-releasing hormone
FKBP5	FK506-binding protein 5
GC	glucocorticoids
GR	glucocorticoid receptors
GR-1F	*NR3C1* GR gene, exon 1F
HPA	hypothalamic–pituitary–adrenal axis
IL	infralimbic ventromedial prefrontal cortex
IML	intermediolateral cell column
LC	locus coeruleus
MAO	monoamine oxidase
MDMA	3,4-methylenedioxymethamphetamine
MR	mineralocorticoid receptor
PFC	prefrontal cortex
PL	prelimbic ventromedial prefrontal cortex
PTSD	post-traumatic stress disorder
PVN	paraventricular nucleus
RVLM	rostral ventrolateral medulla
SABV	sex as a biological variable
SAM	sympathetic–adreno–medullary axis
SCN	suprachiasmatic nucleus
SNS	sympathetic nervous system
SNRI	serotonin–noradrenaline reuptake inhibitor
SSRI	selective serotonin reuptake inhibitor
VH	ventral hippocampus
vmPFC	ventromedial prefrontal cortex
5-HT	serotonin
